# Bilateral Vertebral Venous Sinus Thrombosis Causing Cervical Spinal Cord Compression in a Dog

**DOI:** 10.3389/fvets.2017.00008

**Published:** 2017-02-08

**Authors:** Kathryn E. Rhue, Amanda R. Taylor, Robert C. Cole, Randolph L. Winter

**Affiliations:** ^1^Department of Clinical Sciences, College of Veterinary Medicine, Auburn University, Auburn, AL, USA

**Keywords:** thrombus, tetraparesis, coagulation, warfarin, MRI

## Abstract

A 10-year-old male neutered mixed breed dog was evaluated for cervical hyperesthesia and tetraparesis. Magnetic resonance imaging of the brain and cervical spinal cord identified an extradural compressive lesion over the body of C2 caused by marked dilation of the vertebral venous sinuses. Following intravenous contrast administration both vertebral sinuses had heterogeneous contrast enhancement consistent with incomplete thrombi formation. An abdominal ultrasound also showed a distal aortic thrombus. A definitive cause for the thrombi formation was not identified, but the patient had several predisposing factors which may have contributed. The patient was treated with a combination of warfarin, clopidogrel, and enoxaparin as well as analgesics. Within 48 h of initiation of warfarin therapy, the tetraparesis and hyperesthesia were markedly improved. Repeat abdominal ultrasound 3 weeks after discharge showed reduction in size of aortic thrombus. Neurologic function remained normal for 6 weeks following initiation of treatment. Seventy-four days following initial diagnosis the patient rapidly declined and passed away at home. Necropsy was declined. This is the first report of vertebral venous sinus enlargement leading to spinal cord compression and tetraparesis in a dog. Additionally, warfarin in combination with clopidogrel and enoxaparin appeared to be a safe and effective treatment for the suspected thrombi reported in this case. Vertebral sinus enlargement secondary to thrombi should be considered as a differential diagnosis in patients presenting with tetraparesis and cervical hyperesthesia.

## Introduction

A 10-year-old male, neutered [18.8 kg (41.36 lb)] mixed breed dog was referred for evaluation of a 9-day history of cervical hyperesthesia. Nine days prior to presentation, the dog was trembling and was unwilling to move his neck or shake his head. The following day, the dog presented to his primary care veterinarian where his chronic prednisone (prednisone tablets 10 mg, Qualitest Pharmaceuticals, Huntsville, AL, USA) dosage was increased (from 0.27 mg/kg PO q12h to 0.53 mg/kg PO q12h) and tramadol (tramadol tablets 50 mg, Amneal Pharmaceuticals, Bridgewater, NJ, USA) (2.7 mg/kg PO q12h) was prescribed for cervical hyperesthesia. Due to a lack of improvement after 48 h, the prednisone dose (1.1 mg/kg PO q12h) and tramadol dose frequency (2.7 mg/kg PO q8h) were increased. The dog’s cervical mobility improved, but his cervical hyperesthesia did not improve. The dog was referred for further evaluation.

The dog’s previous history included occasional generalized seizures of unknown etiology beginning 4 years prior to presentation, suspected inflammatory bowel disease, proteinuria [urine protein:creatinine ratio (UPC) 1.25], and decreased hepatic function. The therapy for seizure activity included extended release levetiracetam (levetiracetam extended release tablets 500 mg, Watson Pharmaceuticals, Corona, CA, USA) (106 mg/kg PO q12h), zonisamide (zonisamide capsules 100 mg, Glenmark, Mahwah, NJ, USA) (16 mg/kg PO q12h), potassium bromide (potassium bromide oral solution 250 mg/mL, Letco Medical, Decatur, AL, USA) (33.2 mg/kg PO q24h), topiramate (topiramate tablets, Topamax, 100 mg, Janssen Pharmaceuticals, Raritan, NJ, USA) (10.6 mg/kg PO q8h), gabapentin (gabapentin capsules 300 mg, Ascend Laboratories, Parsippany, NJ, USA) (18.6 mg/kg PO q6h), and clorazepate (clorazepate tablets, 7.5 mg, Mylan Pharmaceuticals Inc., Rockford, IL, USA) (0.8 mg/kg PO q8h during breakthrough seizures). Therapy for the suspected inflammatory bowel disease included a 1-year duration of prednisone use (0.27–0.53 mg/kg PO q12-24h), omeprazole (omeprazole capsules 20 mg, Sandoz Pharmaceuticals, Rotkreuz, Switzerland) (1.1 mg/kg PO q24h), and a hypoallergenic diet [Hill’s Science Diet Z/D (Hill’s Prescription Diet Z/D Dry dog food 25 lb, Hill’s Nutrition, Topeka, KS, USA)]. Hepatic dysfunction was suspected, but not confirmed, to be related to previous long-term phenobarbital use; patient was on phenobarbital for 4 years and this was discontinued 6 months prior to presentation. Therapy for suspected hepatic dysfunction included ursodiol (ursodiol tablets 250 mg, Par Pharmaceutical, Chestnut Ridge, NY, USA) (13.9 mg/kg PO q24h), Denamarin (Denamarin tablets 425 mg, Nutramax Laboratories, Lancaster, SC, USA) (22.6 mg/kg PO q24h), lactulose (lactulose oral suspension 10 g/15 ml, Qualitest Pharmaceuticals, Huntsville, AL, USA) (0.8 mg/kg PO q8h), and milk thistle (milk thistle capsules, 140 mg, NaturesMade, Mission Hills, CA, USA) (dosage unknown) three times daily.

On presentation, the dog was depressed with low head and tail carriage, moderate generalized ataxia/tetraparesis, and a wide-based stance in the pelvic limbs. Femoral pulse strength was mildly decreased bilaterally, and severe hepatomegaly was observed on abdominal palpation. Neurologic exam identified decreased postural reactions in all limbs with notable generalized muscle atrophy and marked cervical hyperesthesia. The cranial nerve exam was normal other than a mild left-sided head tilt, and muscle wasting of the muscles of mastication. Segmental spinal reflexes were normal. The patient was classified as ambulatory tetraparetic with a neuroanatomic localization of spinal cord segments C1–C5 and a modified Frankel score of 4. The CBC was consistent with a stress leukogram, and a serum chemistry panel had elevated liver enzymes (ALKP 3,886 U/L ref 14–152 U/L, alanine transferase 496 U/L ref 13–151 U/L, aspartate aminotransferase 70 U/L ref 18–55 U/L), mild hypoalbuminemia [27 g/L, ref 30–43 g/L (2.7 mg/dL, ref 3.0–4.3 mg/dL)], and hypercholesterolemia [20.27 mmol/L, ref 3.41–8.66 (784 mg/dL ref 132–335)].

**Figure 1:**
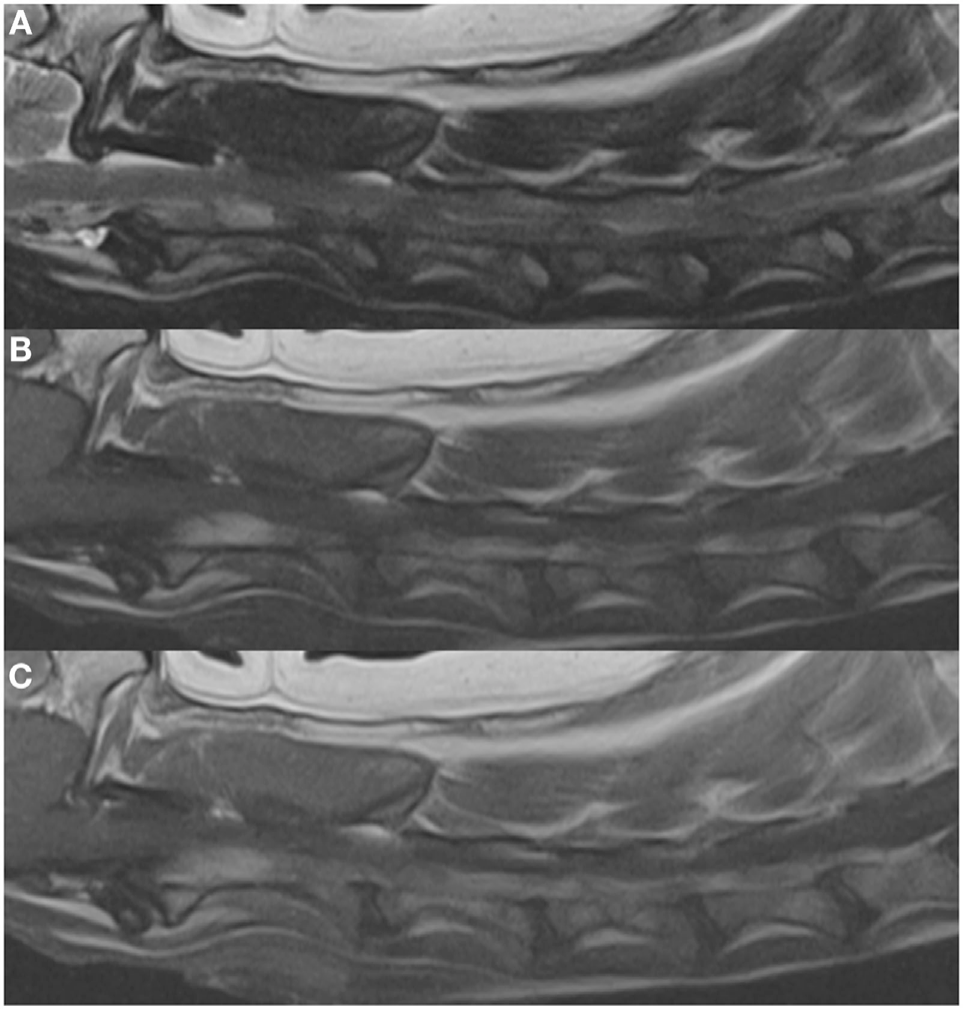
**Left parasagittal images in (A) T2, (B) T1, and (C) T1 + C weighting**. These depict the enlargement of the vertebral venous plexus/sinus resulting in the compression and dorsal elevation of the spinal cord. Enlargement of this plexus is present from the cranial aspect of C2 to the caudal aspect of C6.

**Figure 2:**
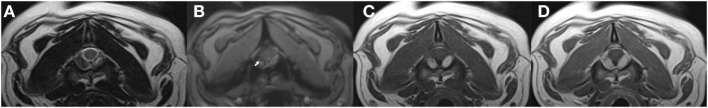
Transverse images at the level of the mid body of C2, in (A) T2, (B) T2*, (C) T1, and (D) T1 + C weighting. The arrow indicates the signal void on the T2* image.

**Figure 3:**
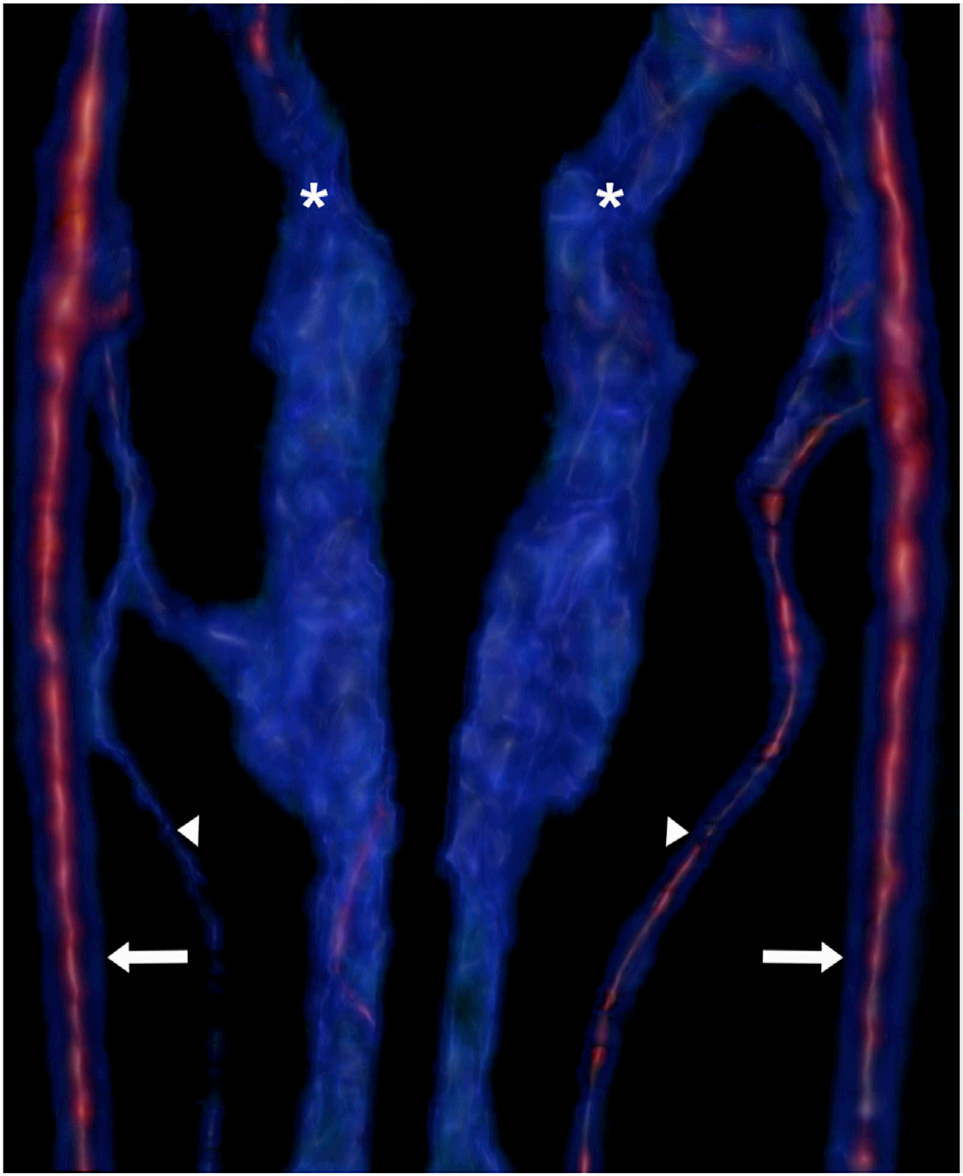
**Reconstruction of the vertebral venous plexus (asterisks), vertebral veins (arrowheads), and internal carotid arteries (arrows)**. The intensity of the red signal indicates contrast enhancement, while the blue signal is less contrast enhanced. The heterogeneity of the blue signal in the plexus indicates heterogenous material in the sinus or turbulent flow.

Imaging studies performed included thoracic and cervical radiography along with magnetic resonance imaging (MRI) (Philips Infinion 1.5T, Philips Medical Systems, Cleveland, OH, USA) of the brain and cervical region performed under general anesthesia. The thoracic and cervical radiographs were considered to be unremarkable for the patient’s age and breed. MRI sequences included T1- and T2-weighted fast spin echo in both sagittal and transverse planes (TR 633–876, TE 98, TR 4,000–8,000, TE 117–118, respectively), transverse T2* (TR 800, TE 23.5), sagittal short T1 inversion recovery (TR 4,000, TE 16.8), sagittal half Fourier acquisition single shot turbo spin echo (TR 40,000, TE 616), postgadolinium (ProHance injectable, 279.3 mg/mL, Bracco Diagnostics, Monroe Township, NJ, USA) (0.1 mmol/kg) T1 fast spin echo in both sagittal and transverse planes (TR 633–876, TE 9.8). Magnetic resonance angiography was acquired using both 2D (TR 5,569, TE 118.3) and 3D time of flight techniques (3D Sliding Interleaved Ky-single interleaved *ky* TR 27, TE 6.7) and reformatted using maximum intensity projection. MRI of the brain and cervical spinal cord identified an extradural compressive lesion over the body of C2 (Figure 1) and extending cranially on both sides of the spinal cord to cranial aspect of C1. On transverse sequences, moderate spinal cord compression was associated with marked dilation of the vertebral venous sinuses (Figure 2). At the mid body of C1, both sinuses were hypointense on T2, and iso to hyperintense on T1 (Figure 2). The same area showed a signal void on T2*. Extending caudally to the mid body of C2 at the site of maximal dilation of the vertebral venous sinuses, the signal pattern was heterogenous on T2*, T2, and more uniform hyperintense signal on T1 (Figure 2). Postgadolinium, there was minimal enhancement noted in the right vertebral venous sinus (Figures 2 and 3). A mild disk protrusion was also noted at C2–C3 with no spinal cord compression. The remainder of the cervical spinal cord and brain were considered to be within normal limits. Based on the moderate spinal cord compression associated with the venous sinus enlargement and the relative lack of compression associated with the disk protrusion at C2–C3, the vertebral sinus enlargement was thought to be the cause of the patient’s clinical signs. The changes in the vertebral sinuses were suggestive of incompletely formed thrombi in both the left and right vertebral sinuses at the level of C1 with subsequent dilation of the sinuses at the level of C2 (Figure 3). The signal pattern of a venous thrombus is similar to that of parenchymal hemorrhage and is dependent on the age of the thrombus. The changes in signal intensity are thought to be related to the paramagnetic effects of hemoglobin breakdown in the thrombus. The T1 hyperintensity and predominantly T2 hypointensity were consistent with a subacute thrombus (6–15 days) (1). Although the changes noted were suggestive of venous thrombosis, slow or turbulent flow can cause a similar signal change on spin echo sequences. Because of this, T2* was acquired in the transverse plane. This sequence has been shown to have higher sensitivity to magnetic susceptibility effects of blood products and can improve detection of intravascular clots (2, 3). The formation of deoxyhemoglobin produces a non-uniform magnetic field with a rapid dephasing of proton spins and subsequent loss of T2* weighted signal as was seen in this case (2, 3). A definitive cause of the suspected thrombi was not identified on MRI.

A lumbar cerebrospinal fluid sample was consistent with albuminocytologic dissociation and minimal blood contamination (0 nucleated cells/μL, 3 red blood cells/μL, protein 92.9 mg/dL). Serum samples were submitted for *Neospora caninum, Toxoplasma gondii, Erhlichia canis, Rickettsia rickettsii*, and *Cryptococcus neoformans* antibody testing as well as aspergillosis EIA antigen testing (Platelia Aspergillus, MiraVista Laboratories, Indianapolis, IN, USA). A urine sample was also submitted for canine distemper virus PCR. The *T. gondii* IgG titer was positive at 1:320 (reference <1:40), while the IgM titer was negative. The aspergillosis EIA galactomannan antigen test was weakly positive (0.77 index; ≥0.5 positive), though it was later noted that the patient had been treated with plasmalyte-A (plasmalyte-A, Baxter Healthcare Corporation, Deerfield, IL, USA) which has been known to cause a false positive with this test (4). Abdominal ultrasonography (Phillips IE33, Phillips Medical Systems, Cleveland, OH, USA) was performed which identified a 1.1 cm × 0.4 cm hyperechoic structure in the distal aorta between the origin of the external and internal iliac arteries with deviation of blood flow around the structure. This was diagnosed as an aortic thrombus. Other findings included marked hepatomegaly with increased echogenicity diffusely and multiple pinpoint hyperechoic foci with numerous ill-defined hypoechoic nodules. Moderate degenerative changes were noted in both kidneys but there was adequate blood flow to each kidney. Echogenic debris was present in the urinary bladder. The changes in the liver were consistent with vacuolar hepatopathy and regenerative nodules with the pinpoint foci representing mineral deposits, though other differentials could not be excluded. No other significant abnormalities were noted.

A urinalysis revealed proteinuria [3,430 g/L (343 mg/dL)], mild hematuria, 10–20 WBC/hpf, and many rod bacteria. A urine culture was positive with heavy growth of *Escherichia coli* (>100,000 col/ml). A coagulation profile had a mild thrombocytosis (538 × 10^9^/L, ref 152–518 × 10^9^/L), minor increase in activated partial thromboplastin time (14.6 s, ref 11.6–14.0), hyperfibrinogenemia [9.70 μmol/L, ref 2.97–4.59 μmol/L (329.9 mg/dL, ref 101–156)], mild decrease in antithrombin (129%, ref >150), and a minor increase in d-dimers (250–500, ref <250). The prothrombin time, plasma fibrin degradation products, and mean platelet volume were within normal reference intervals.

Antithrombotic therapy was initiated with warfarin (warfarin sodium tablets 1 mg, Exelan Pharmaceuticals, Lawrenceville, GA, USA) (0.05 mg/kg PO q24h), clopridogrel (clopidogrel tablets 75 mg, Aurobindo USA, Dayton, NJ, USA) (2 mg/kg PO q24h), and enoxaparin (enoxaparin injection 100 mg/mL, Sandoz Pharmaceuticals, Rotkreuz, Switzerland) (0.8 U/kg SQ q6h). Tramadol was discontinued and the dog was placed on methadone (methadone injectable 10 mg/mL, Mylan Pharmaceuticals Inc., Rockford, IL, USA) (0.2 mg/kg IV q6h), amantadine (amantadine hydrochloride capsules 100 mg, Upsher-Smith Pharmaceuticals, Maple Grove, MN, USA) (2.6 mg/kg PO q12h), and a fentanyl patch (fentanyl transdermal patch 75 μg/h, Apotex, ON, Canada) (4 μg/kg) for pain control. Within 24 h, the methadone was discontinued, and in 48 h the enoxaparin was discontinued. Approximately 48 h following initiation of antithrombotic therapy, the dog’s ataxia markedly improved. Additionally, there were only extremely mild postural reaction deficits present in all four limbs and his femoral pulses were subjectively stronger.

Over the following month, the dog’s neurologic function and treatment remained fairly static other than weekly adjustments to the warfarin dose based on repeated international normalized ratios (INRs). The warfarin dose was changed as previously described, with a goal INR of 2.0–3.0 (5). There were also changes in lactulose administration based on increases in plasma ammonia. Severe proteinuria [3,570–4,910 g/L (357–491 mg/dL)] persisted after resolution of the urinary tract infection. The dog’s pain remained controlled with amantadine and fentanyl; 10 days after initiating treatment, the fentanyl was changed to tramadol. Approximately 3 weeks after initial discharge, a *T. gondii* titer was repeated and was consistent with active infection (increase to 1:1,280), so treatment with clindamycin (clindamycin capsules, 75 mg, 150 mg, Lannett Pharmaceuticals, Philadelphia, PA, USA) (12 mg/kg PO q12h) was initiated. An abdominal ultrasound was also repeated which documented a decrease in the size of the aortic thrombus (0.96 cm × 0.25 cm). The remainder of the ultrasound was otherwise unchanged from the previous exam. An MRI was offered to document any changes in the vertebral venous thromboses, but was declined by the owner.

Over the following weeks, the patient developed worsening of his previously diagnosed hepatic dysfunction which resulted in hepatic encephalopathy. This required increases in lactulose dose and the transient addition of neomycin (neomycin sulfate oral liquid 200 mg/mL, MWI Veterinary Supply, Boise, ID, USA) (9.5 mg/kg PO q12h). Episodes of minor bleeding from the gums and minor wounds developed approximately 6 weeks after initiating warfarin though signs resolved with a decrease in dose. The signs associated with the cervical myelopathy remained resolved 6 weeks after initiation of antithrombotic therapy. On day 74 following diagnosis of the thrombi, the patient was reported to be lethargic and inappetent. Evaluation by a veterinarian was recommended but declined, and the patient passed away at home within 24 h. A necropsy was declined.

Best veterinary care was practiced in the diagnosis and treatment of this patient and owner consent was obtained for all procedures prior to their performance. No experimental protocols were utilized for the diagnosis or treatment of this patient so an institutional review was not required or performed.

## Background

Reports of thromboses causing neurologic dysfunction mostly involve cerebral venous thrombosis (CVT), and these are uncommon in the human literature accounting for <1% of all strokes (6, 7). Vertebral venous thrombosis appears even more rare, with no reports in the veterinary literature and the reports published in the human literature are only vaguely similar to the case reported herein (8–12). Vertebral artery thrombosis, vertebral artery aneurysm, and vertebral arteriovenous malformation with thrombosis have been associated with neurologic dysfunction in human patients (8–10), and chronic myelopathy with concurrent vertebral and spinal artery thromboses in a dog has also been reported (13). Enlargement of the vertebral sinuses have also been associated with neurologic dysfunction and spinal cord compression in human patients (8). One case describes back pain associated with a vertebral sinus hematoma (10), whereas another case series of 13 human patients described radiculopathy and chronic back pain caused by epidural venous enlargement (12). Several factors have been identified as predisposing conditions for central nervous system thromboses including vascular wall injury (8), thrombophilias (6, 7), inflammatory bowel disease (6), head trauma (6), infection (6, 7), vasculitis and other systemic inflammatory diseases (7), cancer (7), and dehydration (6). Vascular malformations (10, 12), iliac or vena caval thrombosis (10, 12), spinal cord compression (10), intracranial hypotension (12), and portal hypertension (12) have been identified as risk factors for epidural venous enlargement. In the veterinary literature, vertebral venous thrombosis has not been reported, though there are sparse case reports of central nervous thromboses associated with meningeal infection (14), head trauma (14), a potential genetic predisposition (13), or idiopathic CVT (14).

This case is the first report of suspected thrombi in the vertebral venous sinuses of a dog leading to clinically significant cervical pain and ambulatory tetraparesis. The patient also had a thrombus in the distal aorta and responded well to antithrombotic therapy including warfarin. Chronic steroid administration, protein-losing nephropathy (PLN), and hepatic dysfunction were considered possible multifactorial causes of the thrombus formation.

## Discussion

Chronic steroid use can create a hypofibrinolytic state (15), damage to the vascular endothelium, hypercoagulability (16), increased platelet activation (16), decreased antithrombin activity (16), and hyperfibrinogenemia (15); all of which may contribute to thrombosis. Dogs with PLN may be hypercoagulable, as 6-22% of dogs with PLN have had a documented thrombotic event (17, 18). The pathogenesis is likely multifactorial (18) with urinary loss of antithrombin (17, 19, 20), platelet hyperaggregability (18, 20), interactions between platelets and fibrin (18), and endothelial cell injury (19) all implicated in this disease process. Based on documentation of an elevated UPC prior to presentation for cervical hyperesthesia, as well as hypoalbuminemia and severity of proteinuria even following resolution of the documented urinary tract infection, the patient was suspected as having a PLN which may also have contributed to thrombosis. Hepatic dysfunction may have also contributed to thrombosis in this dog, as patients with liver disease may be hypercoagulable due to decreased activity of factor Va and factor VIIIa and decreased antithrombin production (21–23). Despite the many coagulation abnormalities associated with hepatobiliary disease, thrombosis is rare and usually precipitated by another factor, such as corticosteroid administration or PLN, shifting the balance of coagulation in favor of thrombi formation (21).

Toxoplasmosis may have contributed to thrombosis formation in this patient. This patient was apparently infected with *T. gondii*, which has been reported to cause cutaneous thrombosis in a single case (24). Even though toxoplasmosis is not typically associated with thrombosis, it is an inflammatory process associated with cell necrosis in various cell types including brain, liver, lungs, and skeletal muscle (25). The contribution of toxoplasmosis if any to the thrombosis is unknown. This patient also had a positive aspergillosis result, which has been reported with cavernous sinus syndrome and CVT in humans (26). Typically dogs with aspergillosis have high titers (>4.0) (27). Based on the mild elevation reported and the previous treatment with plasmalyte-A, the aspergillosis test result is likely a false positive. Ideally a follow-up aspergillosis titer would have been performed 3–4 weeks following discharge from the hospital, but this was declined.

Aortic thrombi are documented in dogs for many causes, but the most common etiologies include hyperadrenocorticism (5, 28), PLN/protein-losing enteropathy (5), hypothyroidism (5), neoplasia (5, 28), and recent steroid administration (5, 28). Warfarin has been reported as a successful treatment for aortic thrombosis in the dog (5) and likely contributed to the improvement in cervical pain and tetraparesis noted, given the positive response within several days of initiating warfarin therapy. Warfarin is a vitamin-K antagonist used for several decades to treat thrombosis in humans (29). Whereas its use in human medicine is frequent, reports of its use in veterinary medicine are rare (5, 29). Several reports (5, 28) have documented warfarin in the treatment of aortic thrombosis with success. To the authors’ knowledge, this is the first report on the use of antithrombotic therapy including warfarin to treat a vertebral venous thrombus in a dog. It is unknown if the episodes of minor bleeding experienced were due to warfarin therapy, or a worsening of the patient’s liver function which was occurring concurrently. Regardless, the bleeding was mild and stopped with a decrease in warfarin dose. Additionally, the patient was receiving clopidogrel which may have contributed to the mild bleeding. Further studies are needed documenting the safety and efficacy of warfarin for the treatment of venous thrombosis.

Although a repeat MRI or necropsy would have identified if the vertebral venous sinus thromboses had resolved, the documented decrease in the size of the aortic thrombus indicated successful antithrombotic therapy. Additionally, the improvement in neurologic function, resolution of ataxia, and resolution of ambulatory dysfunction noted with anticoagulant therapy supports that the vertebral sinus enlargement and subsequent spinal cord compression was caused by bilateral thrombosis. Little information is known regarding the dog’s death, and it is therefore unknown if this was related to neurologic disease. Histopathological confirmation of the presumed vertebral venous and aortic thromboses was not obtained. It is unknown if the vertebral venous enlargement was a condition that preexisted the presumed thromboses of these vessels. Though a definitive cause for the thrombosis in this report was not identified, in up to 58% of cases (5), no concurrent condition is found as a cause of aortic thrombosis.

## Concluding Remarks

Based on the findings in this report, vertebral venous thrombosis should be considered as a cause for cervical hyperesthesia and tetraparesis in dogs predisposed to thrombosis. Additionally, warfarin could be a useful component of therapy for similar cases, though further studies are needed to determine efficacy and treatment length.

## Ethics Statement

This case report describes the clinical management of a dog according to current standard of care. All diagnostic testing and medical management were approved by the dog’s owner.

## Author Contributions

All four contributing authors (KR, AT, RC, and RW) made substantial contributions to the conception or design of the work and the acquisition, analysis, or interpretation of data for the work; they all contributed to the drafting of the work and revising it critically for important intellectual content; they all gave final approval of the version to be published; and they all agree to be accountable for all aspects of the work in ensuring that questions related to the accuracy or integrity of any part of the work are appropriately investigated and resolved.

## Conflict of Interest Statement

The authors declare that the research was conducted in the absence of any commercial or financial relationships that could be construed as a potential conflict of interest.

## Abbreviations

ALKP, alkaline phosphatase; ALT, alanine transferase; aPTT, activated partial thromboplastin time; AST, aspartate aminotransferase; CSF, cerebrospinal fluid; CVT, cerebral venous thrombosis; INR, international normalized ratio; MFS, modified Frankel score; MRI, magnetic resonance imaging; PLN, protein-losing nephropathy; SIRS, systemic inflammatory response syndrome; UPC, urine protein:creatinine ratio; vWF, von Willebrand factor.
